# Developing and Activated T Cell Survival Depends on Differential Signaling Pathways to Regulate Anti-Apoptotic Bcl-x_**L**_

**DOI:** 10.1155/2012/632837

**Published:** 2011-12-26

**Authors:** Ruiqing Wang, Huimin Xie, Zhaofeng Huang, Weirong Shang, Zuoming Sun

**Affiliations:** ^1^Division of Immunology, Beckman Research Institute of the City of Hope, 1500 East Duarte Road, Duarte, CA 91010, USA; ^2^Irell and Manella Graduate School of Biological Sciences, City of Hope, Duarte, CA 91010, USA; ^3^Department of Microbiology and Immunology, Medical School of the University of Illinois, Chicago, IL 60612, USA; ^4^Institute of Human Virology, Zhongshan School of Medicine, Sun Yat-Sen University, Guangzhou, 510080, China; ^5^Department of Gynecology and Obstetrics, Emory University School of Medicine, 550 Peachtree Street, Suite 1800, Atlanta, GA 30308, USA

## Abstract

Survival of T cells in both the central and peripheral immune system determines its ultimate function in the regulation of immune responses. In the thymus, developing T cells undergo positive and negative selection to generate a T cell repertoire that responds to foreign, but not self, antigens. During T cell development, the T cell receptor **α** chain is rearranged. However, the first round of rearrangement may fail, which triggers another round of **α** chain rearrangement until either successful positive selection or cell death occurs. Thus, the lifespan of double positive (CD4^+^CD8^+^; DP) thymocytes determines how many rounds of **α** chain rearrangement can be carried out and influences the likelihood of completing positive selection. The anti-apoptotic protein Bcl-x_L_ is the ultimate effector regulating the survival of CD4^+^CD8^+^ thymocytes subject to the selection process, and the deletion of Bcl-x_L_ leads to premature apoptosis of thymocytes prior to the completion of the developmental process. In addition to its critical function in the thymus, Bcl-x_L_ also regulates the survival of peripheral T cells. Upon engagement with antigens, T cells are activated and differentiated into effectors. Activated T cells upregulate Bcl-x_L_ to enhance their own survival. Bcl-x_L_-mediated survival is required for the generation of effectors that carry out the actual immune responses. In the absence of Bcl-x_L_, mature T cells undergo apoptosis prior to the completion of the differentiation process to become effector cells. Therefore, Bcl-x_L_ ensures the survival of both developing and peripheral T cells, which is essential for a functional immune system.

## 1. Introduction

Bcl-x_L_ is an anti-apoptotic member of the Bcl-2 family of apoptosis regulators. Proteins in this family contain at least one of the four conserved *α*-helical motifs known as Bcl-2 homology (BH) domains (BH1–BH4) [1]. The family members are further classified into three subgroups. The first group contains anti-apoptotic members that possess all four BH domains and includes Bcl-2, Bcl-x_L_, Bcl-w, Mcl-1, Bcl-B, and A1. The other two groups are composed of proapoptotic proteins, which are grouped into those containing three BH domains, including Bax, Bak, and Bok; and those containing BH3 only, including Bad, Bid, Bim, Bmf, Bik, Hrk, Noxa, and Puma. Anti-apoptotic Bcl-x_L_ and Bcl-2 possess a hydrophobic cleft, the BH-3 binding groove, which can accommodate BH3-only members of proapoptotic proteins and neutralize their function [2, 3]. In T cells, Bcl-2 expression is relatively consistent, whereas Bcl-x_L_ expression is induced in response to environment stimuli. In this paper, we will focus on the function and regulation of expression of Bcl-x_L_ in developing and activated mature T cells.

## 2. Bcl-x_**L**_ Function in the Development of T Cells in the Thymus

### 2.1. T Cell Development

T cells are critical components of adaptive immunity, as recognition of foreign antigens by T cells initiates adaptive immune responses. The goal of T cell development in thymus is to arm T cells with all necessary machineries to, upon activation in the periphery, launch responses to foreign antigens by either direct killing (CD8^+^ T cells) or helping other immune cells battle the antigens (CD4^+^ T cells). Therefore, during T cell development, T cells must be educated to target only “nonself” foreign antigens, and this is accomplished by eliminating self-responsive T cells through positive and negative selection in the thymus [4]. T cell development is usually divided into three stages [5]: double negative (DN), double positive (DP), and single positive (SP). At the DN stage, thymocytes express neither CD4 nor CD8, and on the basis of their expression of CD25 and CD44, they are further divided into the DN1 (CD25^−^CD44^+^), DN2 (CD25^+^CD44^+^), DN3 (CD25^+^CD44^−^), and DN4 (CD25^−^CD44^−^) subsets. At the DN3 stage, thymocytes start rearranging the T cell receptor (TCR) *β* chain locus to produce the TCR*β* chain, and only those thymocytes that generate a successfully rearranged TCR*β* chain survive and progress in T cell development. The successfully rearranged TCR*β* chain, combined with the invariant pre-TCR*α* chain, forms a pre-TCR, which delivers signals to stimulate the proliferation of post-*β* thymocytes and instruct the transition from the DN to DP stage. More than 80% of all thymocytes are DP, and only about 5% are positively selected and mature into either CD4^+^ or CD8^+^ SP cells if a DP thymocyte bears a TCR that interacts with an MHC-self peptide complex with sufficiently low affinity. The other DP T cells are negatively selected if the TCR is recognized by an MHC-self peptide with high affinity or die by neglect if the TCR cannot be recognized at all [6]. Once thymocytes mature into SP cells, they migrate out of the thymus into peripheral lymphoid organs, such as the lymph nodes and spleen, to mediate adaptive immunity.

### 2.2. Survival of DP Thymocytes Limits Positive Selection

A critical event, *α* chain rearrangement, is carried out in DP thymocytes. A productive *α* chain rearrangement generates a TCR that recognizes self-MHC, and thus delivers survival signals to allow the T cell to progress to the next stage. However, if *α* chain rearrangement is not productive or produces a TCR that does not recognize self-MHC, T cells can initiate another round of *α* chain rearrangement. DP thymocytes are able to initiate multiple rounds of TCR*α* chain rearrangement until they are either positively selected or die because they have reached the end of their lifespan. Thus, the lifespan of DP thymocytes limits the progression of TCR*α* chain rearrangement and controls the opportunity for assembling a functional TCR [7]. The longer the lifespan of DP cells, the more rounds of rearrangement they can try, and therefore the greater the opportunity for the eventually generation of a TCR that responds to foreign, but not self, antigens. Given the importance of DP thymocyte survival, it is critical that there are precise mechanisms in place to ensure their survival.

### 2.3. Bcl-x_**L**_ Is the Ultimate Survival Factor for DP Thymocytes

The first clue that Bcl-x_L_ regulates DP thymocyte survival was its unique expression pattern during T cell development [8]. During the DN to DP transition, Bcl-x_L_ is specifically upregulated, whereas another survival factor Bcl-2 which belongs to the same family as Bcl-x_L_, is downregulated. Furthermore, Bcl-x_L_ is downregulated while Bcl-2 is upregulated in the following SP stage. The specific upregulation of Bcl-x_L_ during the DP stage strongly suggests that it functions in DP thymocyte survival. Indeed, deletion of Bcl-x_L_led to accelerated apoptosis of DP but not SP thymocytes both *in vitro* and *in vivo* [8, 9], which corresponded to its expression pattern in DP cells. In contrast, overexpression of Bcl-x_L_ led to a significantly increased total thymocyte number due to enhanced DP cell survival [10]. Bcl-x_L_, an anti-apoptotic molecule, is therefore specially upregulated in DP thymocytes to ensure their survival. This then raised the question of what signals are required to stimulate Bcl-x_L_expression in DP cells. Both our work as well as that of others has demonstrated a network of transcriptional factors involved in the regulation of Bcl-x_L_ expression in DP thymocytes. We will discuss each of these factors in the following sections.

### 2.4. ROR*γ*t

ROR*γ*t is a transcription factor that belongs to the steroid nuclear receptor superfamily and was initially identified by expression cloning to screen for molecules that regulate activation-induced cell death [11]. We identified ROR*γ*t by yeast two hybrid screening for CD4 interacting proteins. However, CD4 only binds to ROR*γ*t in yeast, and not in mammalian cells. Similar to Bcl-x_L_, ROR*γ*t is specifically upregulated in DP thymocytes during T cell development, whereas its expression levels are extremely low to undetectable in DN and SP cells. We created ROR*γ*t knockout mice and demonstrated that ROR*γ*t is required for DP thymocyte survival and lymph-node genesis [12], which was confirmed by an independently generated ROR*γ*t knockout mouse strain [13]. ROR*γ*t^−/−^ mice have very small thymuses due to apoptosis of DP thymocytes. The accelerated DP apoptosis was accompanied by greatly reduced Bcl-x_L_ levels, and overexpression of Bcl-x_L_ rescued *ROR*γ*t^−/−^* thymocyte apoptosis, demonstrating that ROR*γ*t enhances DP cell survival by upregulating Bcl-x_L_ expression [12]. We further demonstrated that recruitment of steroid receptor coactivator (SRC) through the activation function 2 (AF2) motif of ROR*γ*t is essential for supporting thymocyte survival by ROR*γ*t [5, 14]. Thus, Bcl-x_L_ was identified as a downstream effector of ROR*γ*t involved in regulation of DP cell survival.

Our recent study also identified TCF-1 as the upstream signaling molecule that regulates the ROR*γ*t-Bcl-x_L_ pathway in DP thymocytes.

### 2.5. TCF-1

TCF-1 is the ultimate effector in the canonical Wnt/*β*-catenin pathway. The Wnt-*β*-catenin pathway has been shown to regulate multiple developmental processes, ranging from regeneration of stem cells to organogenesis of the kidney and reproductive systems [15]. *β*-catenin is usually regulated at the protein level. In the absence of Wnt signaling, several serines and threonines located at the N-terminus of *β*-catenin (amino acids 31–45) are phosphorylated by glycogen synthase-3*β* (GSK-3*β*) bound to the scaffolding proteins axin and adenomatous polyposis coli (APC). The phosphorylated *β*-catenin is a target for ubiquitination and degradation by the 26S proteasome [16]. In addition, there are reports that *β*-catenin can also be degraded in a phosphorylation-independent manner [17, 18]. In the absence of *β*-catenin, TCF-1 is bound by corepressors such as Groucho/Transducin-like enhancer (GRG/TLE) and turns off target gene expression. Activation of Wnt signaling leads to inactivation of GSK-3*β* and accumulation of nonphosphorylated *β*-catenin in the cytoplasm. Accumulated *β*-catenin is then available to bind to and activate TCF-1, which turns on target gene expression.

TCF-1 is important at multiple stages of thymocyte development, including the DP stage. DP thymocytes from *TCF-1^−/−^* mice undergo rapid apoptosis during *in vitro* culture, and thymocyte survival can be restored by expression of full-length TCF-1 but not by truncated TCF-1 that lacks the domain mediating the interaction with *β*-catenin, suggesting that Wnt signaling mediated by *β*-catenin is required to support DP thymocyte survival [19]. To further establish the importance Wnt signaling in DP thymocyte survival, we established a *β*-catenin transgenic mouse strain (**β**-*cat* ^Tg^) that overexpresses constitutively active *β*-catenin under the control of a CD4 promoter [20]. The *β*-catenin transgene is not expressed until the DP stage, which ensures that thymocyte development at DN or earlier stages is not affected. As expected, the four DN subsets have normal distribution and cell numbers in these mice. However, the frequency and numbers of DP thymocytes are significantly greater in **β**-*cat* ^Tg^ mice than in wildtype (WT). In addition, DP thymocytes from **β**-*cat* ^Tg^ mice undergo much slower apoptosis than those of WT mice during both spontaneous and glucocorticoid-induced apoptosis. Furthermore, promotion of DP thymocyte survival by the *β*-catenin transgene is mediated by upregulation of Bcl-x_L_. These data demonstrated that *β*-catenin/TCF-1 extends DP thymocyte survival by up-regulating Bcl-x_L_. However, there was still the question of whether Wnt signaling mediated by *β*-catenin/TCF-1 directly targets Bcl-x_L_ or acts through other factors.

Our recent work has shed light on this by showing that enhancement of DP thymocyte survival by *β*-catenin/TCF-1 is mediated by ROR*γ*t. Microarray analysis revealed that ROR*γ*t was significantly downregulated in *TCF-1^−/−^*  thymocytes that underwent accelerated apoptosis, whereas it was greatly up-regulated in thymocytes that had enhanced survival due to transgenic expression of *β*-*cat* ^Tg^. Both *TCF-1^−/−^* and *ROR*γ*t^−/−^* DP thymocytes underwent similar accelerated apoptosis. Forced expression of ROR*γ*t successfully rescued *TCF-1^−/−^* DP thymocytes from apoptosis, whereas ectopically expressed TCF-1 did not rescue the defective T cell development due to lack of ROR*γ*t-supported survival. Furthermore, activation of TCF-1 by stabilized *β*-catenin could enhance DP thymocyte survival only in the presence of ROR*γ*t, indicating that ROR*γ*t acts downstream of TCF-1 during regulation of DP thymocyte survival. Moreover, *β*-catenin/TCF-1 directly interacted with the ROR*γ*t promoter region and stimulated its activity. Thus, we showed that TCF-1 enhances DP thymocyte survival through transcriptional upregulation of ROR*γ*t, an essential survival molecule for DP thymocytes that acts through upregulation of Bcl-x_L_ [9, 14].

### 2.6. c-Myb

A recent paper by Yuan et al. identified another transcription factor, c-Myb, encoded by the proto-oncogene *Myb*, as an important factor for regulating DP thymocyte survival [21]. In this work, c-Myb was conditionally deleted starting at the DP stage. This deletion led to premature DP thymocyte apoptosis caused by decreased expression of Bcl-x_L_. More specifically, due to an enhanced dependence on Bcl-x_L_ for survival, small preselection DP thymocytes underwent faster premature apoptosis than large preselection and postselection DP thymocytes. Forced expression of Bcl-x_L_ rescued thymocyte survival, and re-introduction of c-Myb restored both Bcl-x_L_ expression and the small preselection DP compartment. The defective DP thymocyte survival caused by reduced expression of Bcl-x_L_ was reminiscent of what has been observed in *TCF-1^−/−^* and *RORγt* ^−/−^ mice. However, the authors proposed that the transcriptional regulation of Bcl-x_L_ by c-Myb is independent of both TCF-1 and ROR*γ*t, since c-Myb expression in both TCF-1- and ROR*γ*t-deficient thymocytes was comparable to that in WT thymocytes, indicating that multiple pro-survival pathways could synergize to ensure proper survival of DP thymocytes via the Bcl-x_L_ pathway.

### 2.7. HEB

HEB is a member of the E protein family. Thymocytes from T lineage-specific HEB-deleted mice undergo rapid apoptosis and have reduced Bcl-x_L_ expression. In c-Myb or ROR*γ*t-deficient thymocytes, forced expression of Bcl-x_L_ rescued DP thymocyte survival, indicating that HEB is another transcription factor that functions upstream of Bcl-x_L_ to promote DP thymocyte survival. In contrast to the independence of ROR*γ*t and TCF-1 in c-Myb-mediated regulation of DP thymocyte survival, HEB regulates ROR*γ*t expression by binding to the two E-box sites present in the ROR*γ*t promoter and stimulating its transcription, which suggests that HEB could act upstream of ROR*γ*t in the same pathway to promote DP thymocyte survival. Since both TCF-1 and HEB are upstream of ROR*γ*t, the relationship between them during the regulation of DP cell survival remains to be determined.

In summary, the transcription factors discussed above work together to form a network for regulating DP thymocyte survival through upregulation of Bcl-x_L_. This complicated network ensures DP thymocytes complete their development in the thymus to generate a functional immune system that responds only to foreign antigens.

## 3. Bcl-x_**L**_ Function during Activation of Peripheral Mature T Cells

### 3.1. T Cell Activation

Adaptive immunity is unique in that only antigen-specific cells are activated to mediate immune responses against specific pathogens. T cells that have just migrated out of the thymus cannot mediate immune responses and therefore are called naïve T cells. Effector T cells differentiated from naïve T cells mediate immune responses *in vivo*. Engagement of TCR by antigen initiates TCR signals that trigger the activation and differentiation of naïve T cells into effector cells, which is an important mechanism for ensuring that only antigen-specific T cells are activated and clonally expanded to become competent effector cells. The T cell activation process is, therefore, not only preparatory to arm T cells for attacking pathogens, but also essential to ensure the adaptive nature of the immune system.

### 3.2. Survival of Activated T Cells Determines Immune Responses

An efficient adaptive immune system must be able to rapidly expand as well as reduce the number of immune cells. T cells meet these requirements, because they can be induced toward proliferation, anergy, or apoptosis depending on the signals received via the TCR. Naïve T cells are activated to proliferate in response to foreign antigens, which is a critical step in adaptive immunity. On the other hand, T cells will undergo apoptosis or anergy if they engage with self-antigens, which is an important mechanism for self-tolerance. Productive engagement of the TCR results in delivery of signals required for T cell proliferation as well as T cell survival. If TCR-mediated survival signals are blocked, T cells undergo apoptosis instead of proliferation upon TCR stimulation. Therefore, TCR-delivered survival signals ensure the completion of the T cell activation process required for differentiation of naïve T cells into effector cells that mediate actual immune responses *in vivo*.

### 3.3. Bcl-x_**L**_ Enhances the Survival of Activated T Cells

Stimulation of the TCR leads to T cell activation, resulting in cell proliferation and production of IL-2. Proliferating T cells, especially during S phase, are susceptible to apoptosis [22, 23]. Thus, TCRs deliver signals to enhance T cell survival during activation [24, 25]. Such survival signals include IL-2, which acts as an extrinsic survival factor. More importantly, activated T cells substantially up-regulate Bcl-x_L_, which intrinsically increases their ability to resist apoptosis [23, 26, 27]. Without Bcl-x_L_, stimulation of T cells via the TCR leads to apoptosis instead of clonal expansion. Therefore, Bcl-x_L_ ensures naïve T cells complete activation. This raises the question of what TCR signals stimulate the upregulation of Bcl-x_L_ during T cell activation.

### 3.4. CD28

CD28, together with its ligands B7.1 and B7.2, is a costimulatory molecule that transduces the secondary signals required for T cell activation. CD28 signaling markedly lowers the TCR signal threshold required for T cell activation, and enhances cytokine production [28]. Another way CD28 facilitates T cell activation is by enhancing intrinsic T cell survival [23, 27, 29]. CD28 costimulation augments the expression of anti-apoptotic Bcl-x_L_, but not that of Bcl-2, to render T cells resistant to apoptosis induced by crosslinking of TCR and Fas, and withdrawal of IL-12 [30]. In contrast to WT T cells, survival of T cells obtained from Bcl-x_L_ transgenic mice is not inhibited by blocking CD28 signals, suggesting that CD28 costimulation sustains T cell survival [29] and that downstream signaling molecules of CD28 are also important for mediating the upregulation of Bcl-x_L_.

### 3.5. PI-3 Kinase

Distinct motifs within the cytoplasmic domain of CD28 regulate T cell proliferation and induction of Bcl-x_L_ [31], suggesting differential signals are responsible for these two CD28-regulated biological effects. PI-3 kinase is required for CD28-mediated induction of Bcl-x_L_, as upregulation of Bcl-x_L_ is prevented by a pharmacological inhibitor of PI-3 kinase and by mutation of the CD28 residues essential for PI-3 kinase activation [31, 32]. Further evidence supporting a role of PI3-kinase in enhancement of T cell survival is that Akt, a target of PI-3 kinase, has been shown to mediate T cell survival by regulating Bcl-x_L_ [33]. Therefore, the PI-3 kinase-Akt pathway mediates CD28 signals to up-regulate Bcl-x_L_ and enhance the survival of activated T cells.

### 3.6. PKC-*θ*

CD28 also facilitates the activation of another important signaling molecule, PKC-*θ*. PKC-*θ* mediates TCR signals essential for T cell activation [34–36] and is required to enhance the survival of activated CD4^+^ T cells by up-regulating Bcl-x_L_. In response to TCR stimulation, CD4^+^ *PKC-*θ*^−/−^* T cells failed to up-regulate Bcl-x_L_ and underwent accelerated apoptosis via a caspase and mitochondria-dependent pathway. Similar to these findings, siRNA-mediated knockdown of PKC-*θ* in Jurkat cells also resulted in apoptosis upon TCR stimulation. Forced expression of Bcl-x_L_ was sufficient to inhibit the apoptosis observed in PKC-*θ* knockdown cells. Furthermore, ectopic expression of PKC-*θ* stimulated a reporter gene driven by a mouse Bcl-x_L_ promoter, whereas the expression of an inactive form of PKC-*θ* or knockdown of endogenous PKC-*θ* led to inhibition of the Bcl-x_L_ reporter. Thus, PKC-*θ*-mediated signals may function not only in the initial activation of naïve CD4^+^ T cells, but also in their survival during T cell activation by directly regulating Bcl-x_L_. PKC-*θ* has a similar function in survival of CD8^+^ T cells [37]. We further demonstrated that PKC-*θ*-regulated survival is essential for cardiac allograft rejection in an adoptive transfer model [38], suggesting that PKC-*θ*-mediated survival plays a role in immune responses *in vivo*.

### 3.7. NF-*κ*B

One of the critical downstream targets of PKC-*θ* is NF-*κ*B. We demonstrated that TCR-initiated NF-*κ*B activation was lacking in *PKC-*θ*^−/−^* T lymphocytes, whereas the activation of NF-*κ*B by tumor-necrosis factor alpha and interleukin-1 was not affected in the absence of PKC-*θ* [36]. Similarly, PKC-*θ* was also found to mediate NF-*κ*B activation in Jurkat cells [39]. There is considerable evidence that TCR-mediated activation of NF-*κ*B extends T cell survival [32, 40], raising the question of whether NF-*κ*B is important for Bcl-x_L_ upregulation. Interestingly, functional NF-*κ*B binding sites are present on the promoter region of Bcl-x_L_ gene [41, 42]. We showed that PKC-*θ*-mediated activation of Bcl-x_L_ promoter was inhibited by dominant negative IKK*β*, suggesting that PKC-*θ* mediates the signals stimulating the expression of Bcl-x_L_ via the NF-*κ*B pathway. Stimulation of the PI-3 kinase/Atk pathway, which enhances T cell survival in a similar manner as PKC-*θ*, leads to activation of NF-*κ*B [40], suggesting that the two pathways may interact in some way during activation of NF-*κ*B. Akt activation is normal in *PKC-*θ*^−/−^* T cells [43, 44], which suggests that Akt is not downstream of PKC-*θ* during activation of NF-*κ*B. There is also no evidence to support that PKC-*θ* is downstream of Akt. Therefore, the current model is that PKC-*θ* and Akt cooperate with each other to mediate the CD28 signals and activate NF-*κ*B, which in turn, stimulates the expression of Bcl-x_L_ required to enhance the survival of activated T cells.

## 4. Summary

Bcl-x_L_ is specifically up-regulated in DP thymocytes during T cell development and in stimulated T cells during T cell activation. This upregulation is important for the completion of T cell development in the thymus as well as the differentiation of naïve T cells into effector cells in the periphery. However, the signaling pathways that regulate Bcl-x_L_ upregulation in the thymus and mature T cells are distinct. In the thymus, a transcription factor network that includes TCF-1, ROR*γ*t, Heb, and c-Myb, which are also important for T cell development, ensures DP thymocyte survival by up-regulating Bcl-x_L_. Whereas in the periphery, CD28-mediated activation of NF-*κ*B via PKC-*θ* and Akt stimulates Bcl-x_L_ expression. Thus, developing and mature T cells use the same factor, Bcl-x_L_, to enhance their survival but through different upstream signaling pathways. Expression of Bcl-x_L_, in contrast to Bcl-2, is inducible and therefore modulates T cell survival in response to environmental signals, which is an essential mechanism for maintaining a functional immune system.
